# An automated deep learning pipeline for EMVI classification and response prediction of rectal cancer using baseline MRI: a multi-centre study

**DOI:** 10.1038/s41698-024-00516-x

**Published:** 2024-01-22

**Authors:** Lishan Cai, Doenja M. J. Lambregts, Geerard L. Beets, Monique Maas, Eduardo H. P. Pooch, Corentin Guérendel, Regina G. H. Beets-Tan, Sean Benson

**Affiliations:** 1 Department of Radiology, The Netherlands Cancer Institute, Plesmanlaan 121, 1066 CX Amsterdam, The Netherlands; 2 GROW School for Oncology and Developmental Biology, Maastricht University Medical Centre, P. Debyelaan 25, 66202 AZ Maastricht, The Netherlands; 3 Department of Surgery, The Netherlands Cancer Institute, Plesmanlaan 121, 1066 CX Amsterdam, The Netherlands

**Keywords:** Cancer imaging, Risk factors

## Abstract

The classification of extramural vascular invasion status using baseline magnetic resonance imaging in rectal cancer has gained significant attention as it is an important prognostic marker. Also, the accurate prediction of patients achieving complete response with primary staging MRI assists clinicians in determining subsequent treatment plans. Most studies utilised radiomics-based methods, requiring manually annotated segmentation and handcrafted features, which tend to generalise poorly. We retrospectively collected 509 patients from 9 centres, and proposed a fully automated pipeline for EMVI status classification and CR prediction with diffusion weighted imaging and T2-weighted imaging. We applied nnUNet, a self-configuring deep learning model, for tumour segmentation and employed learned multiple-level image features to train classification models, named MLNet. This ensures a more comprehensive representation of the tumour features, in terms of both fine-grained detail and global context. On external validation, MLNet, yielding similar AUCs as internal validation, outperformed 3D ResNet10, a deep neural network with ten layers designed for analysing spatiotemporal data, in both CR and EMVI tasks. For CR prediction, MLNet showed better results than the current state-of-the-art model using imaging and clinical features in the same external cohort. Our study demonstrated that incorporating multi-level image representations learned by a deep learning based tumour segmentation model on primary MRI improves the results of EMVI classification and CR prediction with good generalisation to external data. We observed variations in the contributions of individual feature maps to different classification tasks. This pipeline has the potential to be applied in clinical settings, particularly for EMVI classification.

## Introduction

Over the last two decades, advancements in imaging technologies have made stage-specific and personalized treatment of rectal cancer possible^1–4^. Magnetic Resonance Imaging (MRI) is the routine modality used to stratify patients into low, intermediate and high risk groups based on key risk factors such as tumour (T) stage, nodal (N) stage and involvement of the mesorectal fascia^5–7^. In addition, recent guidelines^8^ have also acknowledged extramural vascular (or venous) invasion (EMVI) (see Fig. S2 for EMVI visualisation) as an independent poor prognostic factor that should be taken into account for baseline staging and risk stratification. EMVI is defined as the spread of malignant cells beyond the rectal wall into adjacent perirectal blood vessels and is an important risk factor for local recurrence, distant metastasis and impaired overall survival^9,10^.

In addition to primary staging and risk stratification, MRI also plays an increasingly important role in assessing response to neoadjuvant treatment^11,12^. High-risk (locally advanced) patients typically undergo radiotherapy or combined chemoradiotherapy (CRT) to induce tumour downsizing and downstaging prior to surgery. As a result of CRT, up to 27% of patients may achieve a complete response (CR)^13^. Organ-preserving (watch and wait) treatment may be offered as an alternative to standard resection for these patients, provided that they can be accurately selected. This option has been associated with favourable long-term oncological outcomes and improved quality of life^14^. The combination of digital rectal examination, endoscopy and MRI including diffusion-weighted imaging (DWI) has been shown to yield good diagnostic performance to identify a CR after completion of CRT^15^. In addition to assessing response after completion of CRT, recent studies^16^ have focused on early response prediction using imaging biomarkers derived from baseline MRI (including DWI) scans. Predicting response before the start of treatment could create new opportunities to further personalise neoadjuvant treatment schemes depending on the anticipated response. Recent studies^17–20^ demonstrate reasonable results for predicting risk factors such as EMVI and response to CRT by combining Artificial Intelligence (AI) techniques with MRI to develop prognostic image biomarker models. So far, these models have mostly used combinations of clinical and/or radiomics features, which require MRI manual delineation, feature extraction, and feature selection steps. Ao et al. ^17^ assessed preoperative EMVI using quantitative Dynamic Contrast-Enhanced MRI and DWI parameters, achieving an area under the ROC curve(AUC) of 0.856 with 84 patients from a single centre. Shu et al. ^18^ proposed an EMVI prediction model using multiparametric MRI including T2-weighted images (T2W), T1-weighted images (T1W), and DWI, with an AUC of 0.835 on 317 patients from a single-centre dataset without an external validation.

Regarding CR prediction, Bourbonne et al. ^21^ have concluded in their recent review that substantial efforts have been made to improve the quality of published radiomics models. As of the 14th of November 2022, there were 36 studies concerned MRI-only radiomics with reported AUC ranging from 0.70 to 0.95, and most were retrospective studies based on pre-CRT only MRIs. Also, delineation of the tumour volumes was manually done by radiologists in most studies, which hinders the implementation of fully automated classification models. Some studies applied deep learning (DL) techniques. Unlike radiomics using handcrafted and quantifiable features, DL is able to extract features automatically from images. Zhu et al. ^22^ proposed a DL model to predict response by training with Apparent Diffusion Coefficient (ADC) patches delineated by radiologists. Their DL model achieved an AUC of 0.851 (95% CI: 0.789–0.914), again based on data from a single centre. Jin et al. ^23^ presented a multi-task deep learning approach consisting of two Siamese sub-networks that are joined at multiple layers. The multi-task model utilises both pre and post-treatment multiparametric MRI (DWI, T2W, T1W, T1-weighted with contrast-enhancement (T1W + C)), achieving an AUC of 0.95 in two independent cohorts. However, the same model was trained by Wichtman et al. ^24^ in a multi-centre (4 centres) scenario. Their model showed an AUC of 0.60 when using the combination of pre and post-therapeutic T2W, DWI, and ADC maps as input. Wichtmann et al. ^24^ demonstrated the current challenge of constructing deep learning models using multi-institutional medical data. Data from different origins can contain significant variations based on specific parametrisation, creating a domain shift problem observed in multiple medical imaging modalities^25,26^.

In the management of rectal cancer using AI, there is a lack of multi-centre studies to validate the generalisability of the models and their feasibility for automated implementation in clinical settings. In this study, we introduced a fully automated deep learning pipeline. The pipeline consists of nnUNet^27^, a self-configuring DL tumour segmentation model and a classification model utilising multi-level image representations learned by nnUNet, named as MLNet. To validate the pipeline, we used a multi-centre dataset including data from 509 patients from 9 medical centres in the Netherlands. The proposed automated pipeline aims to classify EMVI status and predict treatment response using primary staging MRI further to provide potential additional value to the preoperative clinical workflow.

## Results

### Characteristics of cases

We used a dataset collected as part of a previously published multi-centre study, which included the baseline staging MRI (DWI and T2W) of 509 patient cases (obtained from one university hospital, seven large teaching hospitals and one comprehensive cancer centres from Southeren and Northern part of the Netherlands) with locally advanced rectal cancer undergoing neoadjuvant CRT. Further in- and exclusion criteria were according to those described by Schurink et al. ^28^. Baseline T and N staging variables cT-stage (cT1-2, cT3, cT4), cN-stage (cN0, cN1, cN2) were derived from the original staging reports that were performed by a multitude of readers. The data were grouped into mrEMVI+ and mrEMVI- cases, based on clinical assessment by an expert radiologist (D.M.J.L.) with >10 years of dedicated experience in rectal MRI. In total, there were 304 EMVI+ cases and 205 EMVI- cases. Additionally, the data was divided into pathological complete response of the primary tumour (CR) and non-complete response (non-CR) groups. CR was defined as either a complete pathological response after surgery (pCR = ypT0) or a sustained clinical complete response (cCR) with no evidence of a luminal regrowth on repeated follow-up MRI and endoscopy for a period of longer than 2 years. There were 368 cases of non-CR and 141 cases of CR. Lymph nodes were not taken into account. The characteristics of rectal cancer cases used in our study are summarised in Table 1. There were no significant differences among the basic demographic features and tumour characteristics of the development cohort and external validation cohort (all *p* values > 0.05).

**Table 1:** Summary of patient demographic and clinical characteristics of the multi-centre dataset.

		All	Development Train Internal Val		External Val	*p* value
Age (median, range)		65 (25−87)	66 (25−87)	64 (39−85)	65 (33−81)	0.37
Gender	Female Male	177 (35%) 332 (65%)	104 (33%) 214 (67%)	36 (37%) 59 (62%)	38 (39%) 59 (61%)	0.31
cT	1−2 3 4	35 (7%) 441 (81%) 60 (12%)	22 (7%) 259 (82%) 36 (11%)	8 (8%) 75 (79%) 12 (13%)	5 (5%) 80 (83%) 12 (12%)	0.56
cN	0 1 2	68 (13%) 122 (24%) 319 (63%)	34 (11%) 66 (21%) 217 (68%)	18 (19%) 37 (39%) 40 (42%)	16 (16%) 19 (20%) 62 (64%)	0.98
Response	non-CR CR	368 (72%) 141 (28%)	227 (71%) 91 (29%)	75 (79%) 20 (21%)	67 (69%) 30 (30%)	0.43
EMVI	EMVI + EMVI −	304 (60%) 205 (40%)	204 (64%) 114 (36%)	50 (53%) 45 (47%)	50 (52%) 47 (48%)	0.07
Total		509	317	95	97	

Values in age parentheses are the minimum and maximum. Values in parentheses of other items are the percentages. EMVI + : EMVI positive. EMVI-, EMVI negative. *P* values were calculated using the Kruskal-Wallis test between the development cohort and the external validation cohort.

*cT* baseline T staging, *cN* baseline N staging, *non-CR* non-responders, *CR* complete responders.

### Tumour segmentation

**Fig. 1:**
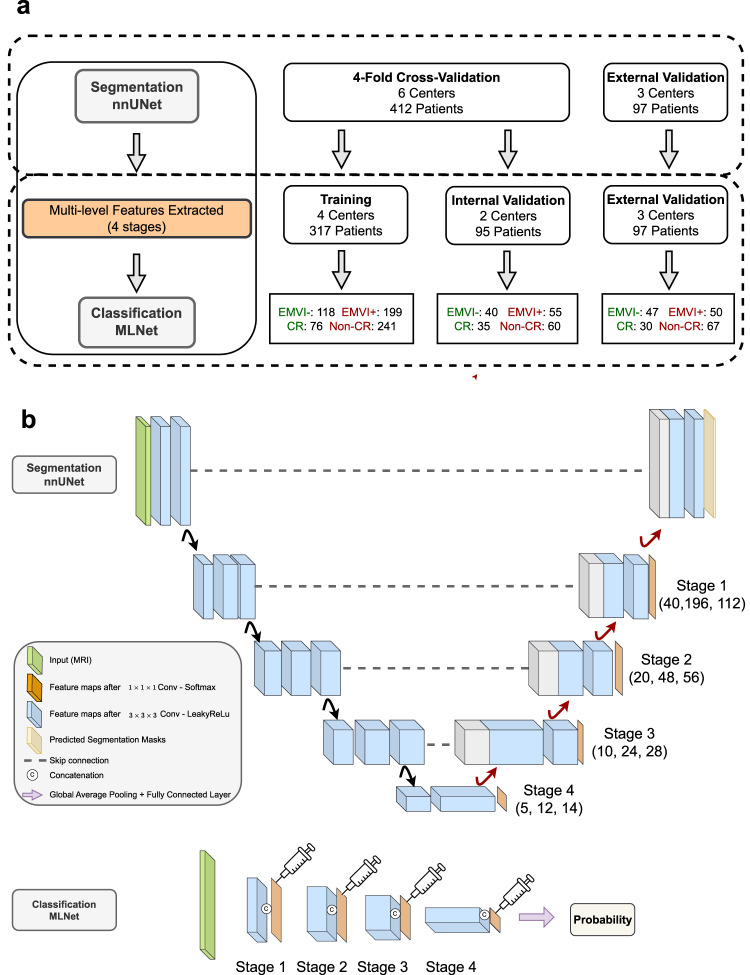
Workflow diagram. **a** the experiment workflow. For the rectal tumour segmentation, 4-fold cross-validation was done with DWI or DWI + T2W from 6 centres, 412 patients. For classification tasks, 4 out of these 6 centres’ data were used as the training and 2 centres were internal validation. The other 3 centres were external validation for both segmentation and classification tasks. **b** The automated pipeline containing segmentation and classification models: Image feature maps from different stages (orange, stage 1 to stage 4) were inferred from rectal tumour segmentation nnUNet. The inferred multi-level features (orange) were then injected by concatenation in different levels of the MLNet, where 3D ResNet10 was used as a backbone without skip connections, to assist the classification tasks. The original MRI was used as input for MLNet (Green).

**Fig. 2:**
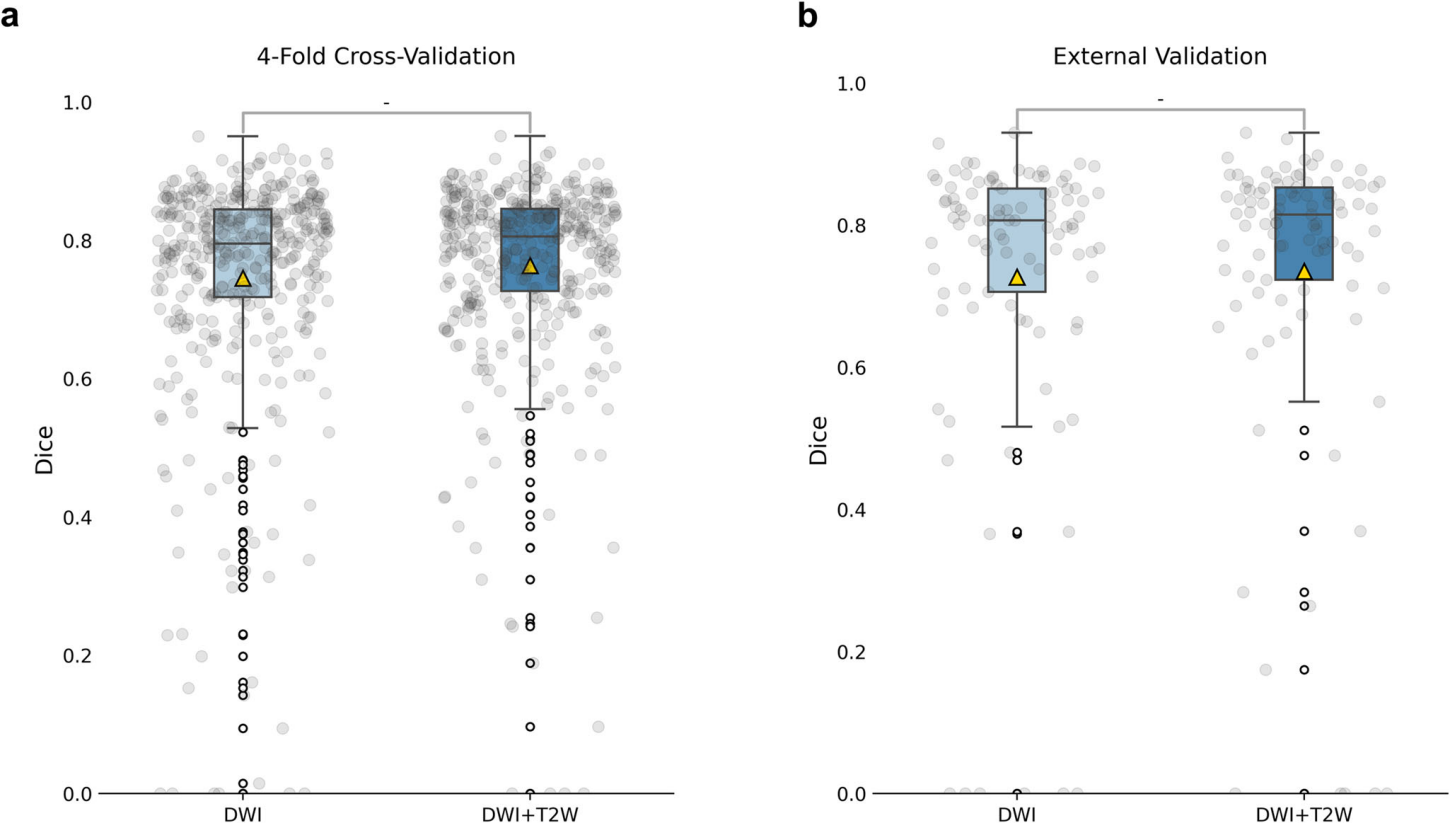
The segmentation performance using nnUNet in the internal and external cohorts. **a** The boxplot of rectal tumour Dice on 4-fold cross-validation. **b** The boxplot of rectal tumour Dice on External validation. The top and bottom edges correspond to the 75th and 25th percentiles (Q3 and Q1), respectively. The line inside the box represents the median value (50th percentile). The yellow triangle denotes the mDice. The whiskers in the box plot extend to 2 times the interquartile range (IQR).

**Fig. 3:**
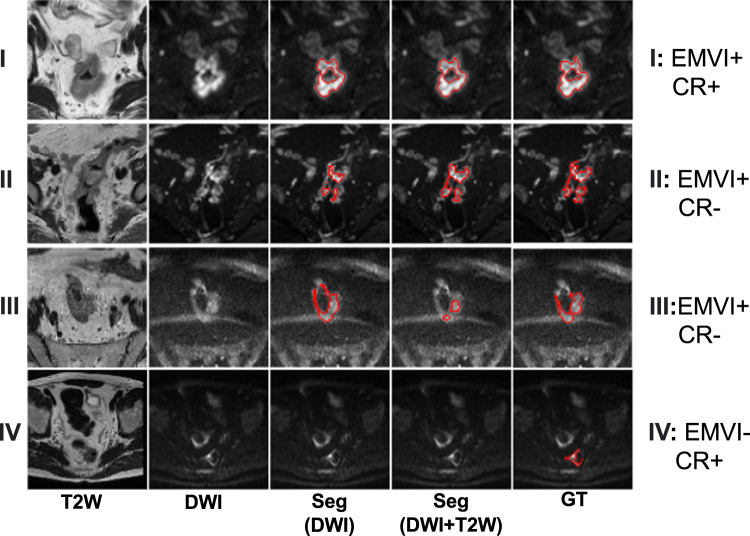
The visualization of four predicted segmentation from the external cohort. The rows **I−IV** were different segmentation cases from the external cohort. Columns from left to right represent the T2W slices, DWI slices, predicted segmentation masks from DWI nnUNet, predicted segmentation masks from DWI + T2W nnUNet and ground truth masks. nnUNet showed good performance on **I**, with dice 0.93 for both DWI and DWI + T2W. For case **II**, nnUNet with DWI + T2 has better segmentation ability but DWI alone showed a better result in case **III**. Both nnUNet models fail on the prediction of case **IV**.

In the first part of our proposed automated pipeline, see Fig. 1, we trained 2 nnUNet models with DWI and DWI + T2W separately. Dice similarity score (Dice) was used to measure the segmentation performance. The mean Dice (mDice) of the 4-fold cross-validation from DWI and DWI + T2W were 0.75 and 0.76 respectively. The mDices of external validation were 0.73 (DWI) and 0.74 (DWI + T2W), see Table 2. By adding T2W, the mDices for both cross-validation and external validation increased by 1%. The Dice difference between DWI and DWI + T2W segmentation is not significant with *p* > 0.05 (*p* = 0.31 for internal validation, *p* = 0.61 for external validation). From the boxplot in Fig. 2b, nnUNet had trouble with segmenting some cases (with Dice < 0.20) and failed to delineate several hard samples (Dice = 0.00). Figure 3 is the illustration of the segmentation performance for cases **I−IV** from external data and their corresponding Dice can be found in Table 2.

**Table 2:** Segmentation results using nnUNet.

	4-fold (STD)	External (STD)	I	II	III	IV
DWI	0.75 (0.17)	0.73 (0.21)	0.93	0.57	0.66	0.0
DWI + T2W	0.76 (0.14)	0.74 (0.22)	0.93	0.79	0.21	0.0

mean Dice similarity Score (mDice) was used to measure the overall segmentation performance. STD, standard deviation. Cases **I−IV** from external validation.

**Fig. 4:**
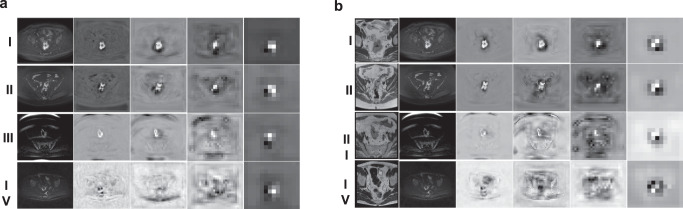
Feature maps visualization. Feature maps derived from nnUNet by deep supervision cases for **I−IV**. **a** using DWI alone; (**b**) using DWI + T2W.

After the training of tumour segmentation, 4-stage feature maps derived from nnUNet were inferred. The visualization of feature maps from different stages for case **I−IV** can be seen in Fig. 4. Stages 1−2 represented more superficial, finer features and stages 3−4 showed coarser, more abstract image representations. nnUNet failed to delineate the rectal tumour for case **IV** with Dice 0.00, but feature maps were able to capture the tumoural regions.

### EMVI classification and Complete Response prediction

**Fig. 5:**
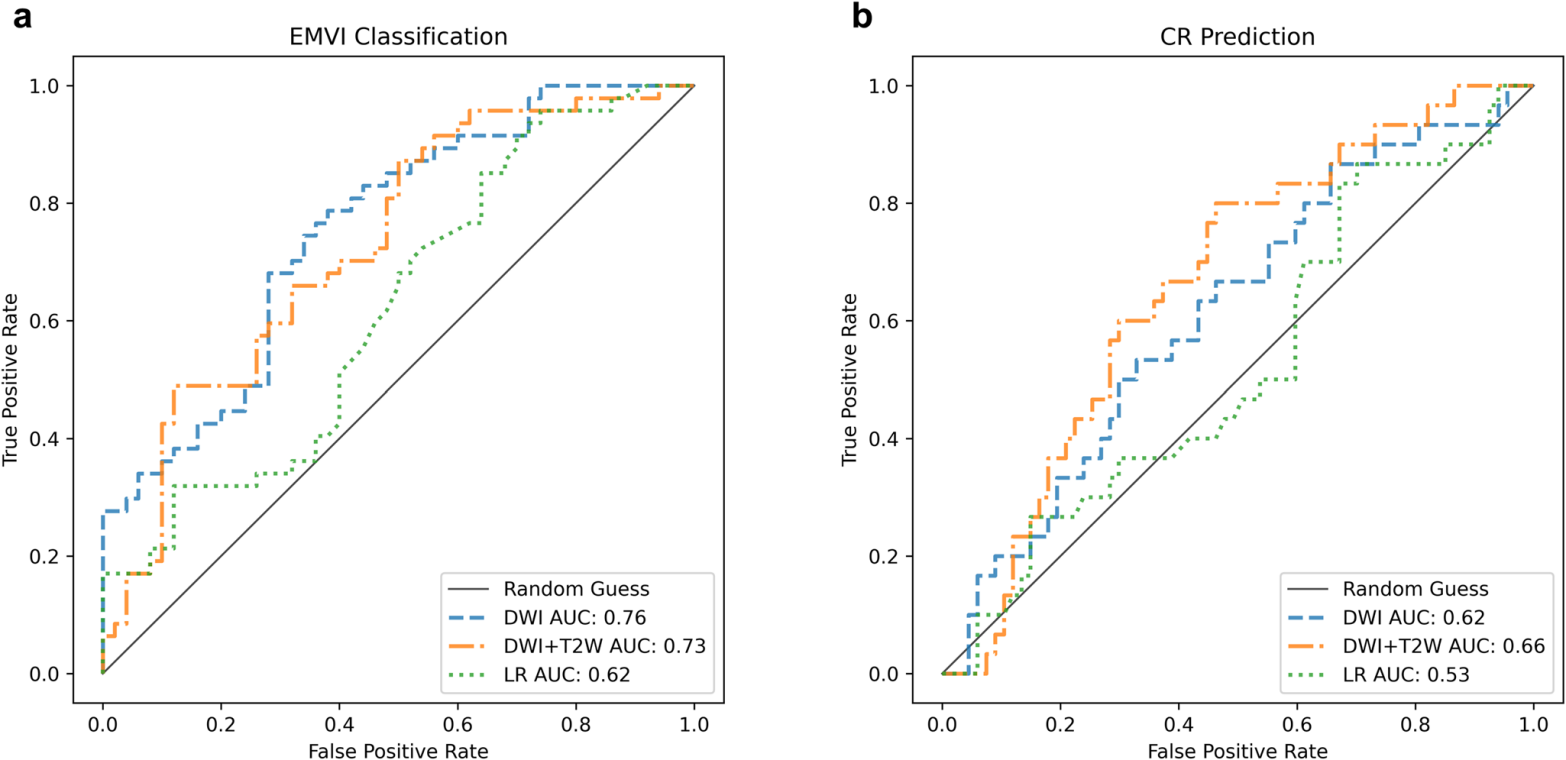
The ROC Curves for EMVI classification and CR prediction in the external cohort (*n* = 97). The receiver operating characteristics (ROC) plots using DWI or DWI + T2W with MLNet and multivariate analysis using logistic regression. **a** EMVI classification (**b**) CR prediction.

Tables 3–4 and Fig. 5 showed EMVI classification and CR prediction results using MLNet with DWI only and DWI + T2W on the external validation. Multivariate analysis was also done for both EMVI and CR tasks using logistic regression to compare the predictive effects of clinical factors including age, gender, T and N staging, with MLNet. For EMVI classification, the MLNet with DWI alone showed better classification power with AUC 0.76 (0.66−0.84) (Table 3, Fig. 5a) in the external validation and AUC 0.76 (0.66−0.85) in the internal validation (Table S4). The addition of T2W resulted in an increase of the internal AUC to 0.78 (0.68−0.87) as indicated in (Table S6). However, the external AUC exhibited a decline to 0.73 (0.62−0.83), suggesting signs of overfitting. Nevertheless, with respect to the prediction of CR, the combination of DWI and T2W demonstrated superior performance, yielding an AUC of 0.66 (0.55−0.77) in the external cohort (Table 4, Fig. 5b) and 0.65 (0.52−0.77) in the internal validation set (Table S7). These results outperformed the utilisation of the DWI-only pipeline, which produced an external AUC of 0.62 (0.49−0.73) and an internal AUC of 0.62 (0.50−0.74) (Table S5). MLNet demonstrated superior performance for both EMVI and CR tasks in comparison to the multivariate analysis in the external cohort and development cohort (Tables S8−10).

**Table 3:** EMVI classification results in the external cohort.

Network	AUC (95% CI)	Sensitivity(95% CI)	Specificity(95% CI)	PPV (95% CI)	NPV (95% CI)	F1 (95% CI)
LR	0.62 (0.51−0.73)	0.55 (0.41−0.70)	0.56 (0.42−0.70)	0.54 (0.40−0.68)	0.57 (0.43−0.71)	0.55 (0.42−0.67)
DWI	0.76 (0.66−0.84)	0.67 (0.55−0.76)	0.70 (0.59−0.80)	0.67 (0.52−0.80)	0.69 (0.55−0.80)	0.67 (0.54−0.78)
DWI + T2W	0.73 (0.62−0.83)	0.63 (0.53−0.73)	0.67 (0.57−0.77)	0.64 (0.50−0.77)	0.67 (0.52−0.78)	0.62 (0.52−0.74)

In the parentheses are 95% confidence intervals (95% CI). LR, Logistic regression using clinical factors. DWI, pipeline only using DWI. DWI + T2W, pipeline using both DWI and T2W. The best metrics were highlighted in bold.

**Table 4:** CR prediction results in the external cohort.

Network	AUC (95% CI)	Sensitivity(95% CI)	Specificity(95% CI)	PPV (95% CI)	NPV (95% CI)	F1 (95% CI)
LR	0.53 (0.40−0.65)	0.37 (0.19−0.55)	0.61 (0.49−0.73)	0.29 (0.15−0.45)	0.68 (0.56−0.80)	0.33 (0.18−0.47)
DWI	0.62 (0.49−0.73)	0.56 (0.44−0.68)	0.60 (0.49−0.71)	0.39 (0.24−0.54)	0.75 (0.63−0.86)	0.46 (0.32−0.59)
DWI + T2W	0.66 (0.55−0.77)	0.61 (0.49−0.72)	0.65 (0.54−0.75)	0.44 (0.28−0.60)	0.79 (0.67−0.88)	0.51 (0.36−0.64)

In the parentheses are 95% confidence intervals (95% CI). LR, Logistic regression using clinical factors. DWI, pipeline only using DWI. DWI + T2W, pipeline using both DWI and T2W. The best metrics were highlighted in bold.

**Fig. 6:**
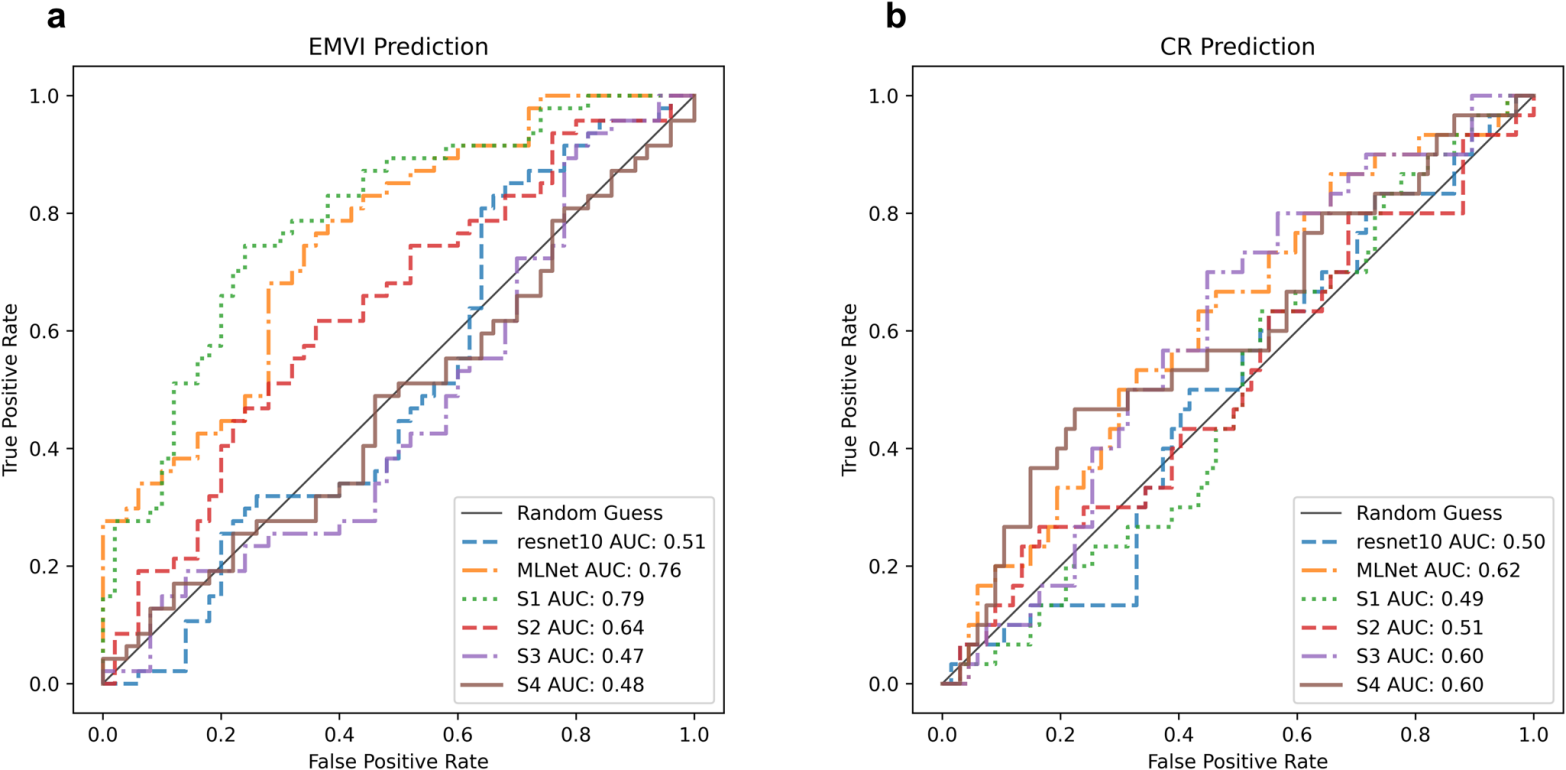
The ROC Curves for the ablation study of EMVI classification and CR prediction in the external cohort (*n* = 97) using DWI only. The ROC plots of (**a**) EMVI classification (**b**) CR prediction. S1(2,3,4): classification network only infused segmentation features from stage1 (2, 3, 4).

Tables 5–6 and Fig. 6 showed the ablation analysis of the EMVI classification and CR prediction from ResNet10, MLNet and the individual stage of feature maps solely using DWI in the external cohort and the results for the internal cohort can be found in the Tables S4–5. In the case of EMVI classification (Table 5, Fig. 6a), it was observed that features extracted from the first and second stages, which encompassed finer details and more information-rich attributes, played a more pivotal role in the model’s decision. Particularly, the network solely utilizing features from the first stage achieved noteworthy performance, yielding an AUC of 0.79 (0.70−0.87), surpassing MLNet’s performance, which incorporated representations from all four stages and achieved an AUC of 0.76 (0.66−0.84). In contrast, for CR prediction (Table 6 Fig. 6b), features from the third and ourth stages, characterised by coarser semantic attributes, had a more substantial impact on the final decision. Nevertheless, MLNet exhibited the best performance in the CR task. Similar patterns were also observed in the ablation analysis using both T2W and DWI, see Tables S2–3, Fig. S3 for the external validation and Tables S6−7 for the internal validation.

**Table 5:** EMVI classification ablation study using DWI only in the external cohort.

Network	AUC (95% CI)	Sensitivity(95% CI)	Specificity(95% CI)	PPV (95% CI)	NPV (95% CI)	F1 (95% CI)
ResNet10	0.51 (0.39−0.62)	0.44 (0.34−0.55)	0.48 (0.38−0.59)	0.45 (0.31−0.60)	0.48 (0.34−0.62)	0.44 (0.33−0.56)
MLNet	0.76 (0.66−0.84)	0.67 (0.55−0.76)	0.70 (0.59−0.80)	0.67 (0.52−0.80)	0.69 (0.55−0.80)	0.67 (0.54−0.78)
Stage 1	0.79 (0.70−0.87)	0.72 (0.62−0.81)	0.75 (0.66−0.84)	0.73 (0.60−0.85)	0.74 (0.61−0.84)	0.72 (0.61−0.82)
Stage 2	0.64 (0.52−0.74)	0.59 (0.49−0.69)	0.63 (0.52−0.73)	0.60 (0.45−0.73)	0.62 (0.48−0.75)	0.60 (0.48−0.70)
Stage 3	0.47 (0.35−0.59)	0.42 (0.32−0.53)	0.46 (0.36−0.56)	0.43 (0.29−0.57)	0.46 (0.32−0.60)	0.42 (0.31−0.54)
Stage 4	0.48 (0.36−0.60)	0.47 (0.36−0.58)	0.51 (0.40−0.62)	0.47 (0.33−0.62)	0.50 (0.36−0.65)	0.47 (0.35−0.59)

Values in the parentheses were 95% confidence intervals (95% CI). Stage1(2,3,4), classification network only infused segmentation features from stage1 (2,3,4). The best metrics were highlighted in bold.

**Table 6:** CR prediction ablation study using DWI only in the external cohort.

Network	AUC (95% CI)	Sensitivity(95% CI)	Specificity(95% CI)	PPV (95% CI)	NPV (95% CI)	F1 (95% CI)
ResNet10	0.50 (0.38−0.62)	0.50 (0.37−0.61)	0.53 (0.43−0.65)	0.32 (0.20−0.47)	0.71 (0.57−0.82)	0.39 (0.26−0.52)
MLNet	0.62 (0.49−0.73)	0.56 (0.44−0.68)	0.60 (0.49−0.71)	0.39 (0.24−0.54)	0.75 (0.63−0.86)	0.46 (0.32−0.59)
Stage 1	0.49 (0.37−0.62)	0.46 (0.34−0.58)	0.51 (0.41−0.61)	0.29 (0.18−0.43)	0.68 (0.54−0.80)	0.36 (0.24−0.49)
Stage 2	0.51 (0.39−0.64)	0.47 (0.35−0.59)	0.51 (0.41−0.62)	0.30 (0.18−0.44)	0.69 (0.55−0.81)	0.36 (0.24−0.49)
Stage 3	0.60 (0.48−0.71)	0.56 (0.43−0.67)	0.60 (0.49−0.70)	0.38 (0.24−0.54)	0.75 (0.63−0.85)	0.45 (0.32−0.59)
Stage 4	0.60 (0.47−0.72)	0.53 (0.40−0.67)	0.57 (0.44−0.70)	0.36 (0.20−0.52)	0.73 (0.59−0.85)	0.43 (0.27−0.57)

Values in the parentheses were 95% confidence intervals (95% CI). Stage1(2,3,4), classification network only infused segmentation features from stage1 (2,3,4). The best metrics were highlighted in bold.

### AI explainability

**Fig. 7:**
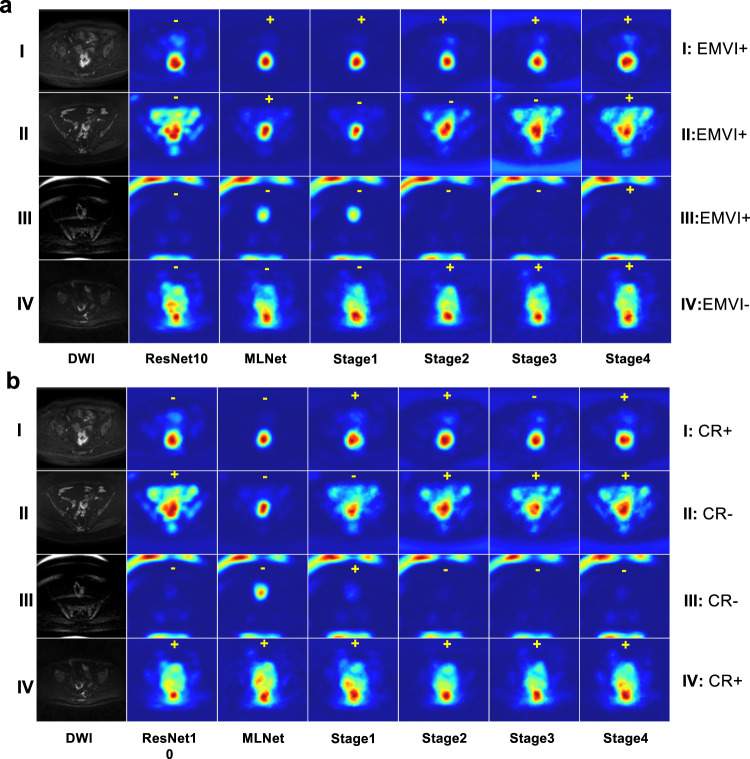
The visualization based on the Grad-CAM + + method of ablation studies for EMVI classification and CR prediction. **a** The visualization for EMVI classification. The ground truth EMVI statuses were on the left side **I**: EMVI + ; **II**: EMVI + , **III**: EMVI + ; **IV**: EMVI-. The annotated “+“ or “-“ on the attention maps were predicted EMVI classification of corresponding models. **b** The visualization for CR prediction. The ground truth response outcomes were on the left side. **I**: CR + , **II**: CR-, **III**: CR-, **IV**: CR + . The annotated “+“ or “-“ on the visualization maps were CR predictions of corresponding models.

To explore the interpretability of classification models, we showed the attention maps of networks in ablation analysis for both EMVI and response using Grad-Cam + +^29^ (see Fig. 7). In case **I**, all the classification networks including 3D ResNet10 successfully concentrated on the tumuoral and surrounding regions for both tasks. In cases **II** and **III**, MLNet effectively guided its attention to the tumour and peri-tumour areas for both EMVI and CR experiments. While using features exclusively from the first stage, the model exhibited a selective focus solely on tumour-related areas during the EMVI classification, failing to encompass the same focus in the CR task. Furthermore, with the progression to coarser features (stages 3−4), the network lost its ability to focus on the tumour. For cases **II** and **III**, in the response prediction task, the model with features from a single stage alone appeared to be limited in guiding the model to concentrate on rectal tumour regions. MLNet highlighted tumour and peri-tumoural areas in case **IV**, despite the failure of tumour segmentation (Dice = 0.00). Overall, we observed that by injecting four-stage feature maps from segmentation networks, MLNet was guided to be able to effectively focus on tumoural and peri-tumoural regions for classification tasks across all four cases. In some specific cases, features from a single stage alone were also capable of localising the tumour and its neighbouring regions.

## Discussion

We have proposed a fully automated pipeline for rectal tumour segmentation, the classification of EMVI status and the prediction of the treatment outcome (complete response to CRT). The pipeline consists of nnUNet and MLNet, a lightweight CNN. nnUNet was trained to achieve automated tumour segmentation and extract different scale features from baseline MRI. MLNet, which fuses inferred segmentation features into 3D ResNet10, was trained to classify EMVI status and to predict treatment response. The nnUNet model demonstrated favourable rectal tumour segmentation performance and generalisation capabilities, as evidenced by achieving a mDice of 0.73 (0.74) on external validation and 0.74 (0.76) on cross-validation using DWI (DWI + T2W) in multi-centre background.

For EMVI classification, the performance was 0.76 (0.66−0.84) on the external validation dataset. With only the finest feature map (stage 1), the AUC of external validation could reach up to 0.79 (0.70−0.87) using only DWI. For CR prediction, MLNet achieved AUC of 0.66 (0.55−0.77), outperforming the current state-of-the-art by Schurink et al. ^28^ on the same external cohort. Schurink et al. ^28^ developed a clinical-imaging model to predict CR. The best-performing model, using non-imaging (weeks to surgery) and advanced staging variables (tumour height, T and N staging, invasion depth and tumour length), achieved an AUC of 0.60 (0.53−0.76). Like other Radiomics-based or tumour-centre crop-based models, one limitation of the study from Schurink et al. ^28^ was that manually annotated segmentation for both development and test cohorts by experienced radiologists was required. To solve this, Jin et al. ^23^ have proposed a multi-tasking learning model with pre-and-post multiparametric MRI for both segmentation and pCR assessment and the model shows the state-of-the-art results with single-centre data. The drawback of such a multi-tasking network is that firstly, it requires both pre- and post-MRIs. Pre-treatment prediction of response is potentially beneficial for personalising neoadjuvant strategies. Also, it is better to visualise the rectal tumour in pre-treatment than post-treatment MRIs, where high signal areas are frequently less noticeable and may be distributed throughout the fibrosis^30^. Secondly, the training of such a heavy multitasking model is computationally expensive. In our study, only baseline MRI was used. Training and inference of lightweight MLNet were significantly faster than the multitasking network. Some studies have also proposed machine learning based automated workflows. Defeudis et al. ^31^ have demonstrated automated pCR radiomics models after nCRT in LARC using DWI and T2W performed before CRT. The AUC could reach 0.81 (0.60–0.89) over external validation data. However, the main limitation is that they have excluded all cases with automated segmentation dice lower than 0.2 as they cannot guarantee that radiomics features are from the targeted tumour regions with such poor segmentation results, where prediction AUC is biased to high Dice cases. In the multi-centre background, predicting masks with dice lower than 0.2 could often occur due to data heterogeneity. MLNet solved this problem by injecting tumour representations derived from different levels of nnUNet. For instance, in the case of **IV**, even though the segmentation network failed to contour the rectal tumour, MLNet was capable of capturing hidden features for CR prediction.

Most radiomics models were only looking at tumour core regions. However, peri-tumoural regions also potentially contain useful information. Delli Pizzi et al. ^32^ presented an MRI-based machine learning model using clinical features and radiomics features extracted from both “tumour core” (the whole rectal tumour manually segmented on pre-treatment T2W) and “tumour border” (the most peripheral portion of the tumour core and the surrounding tissues). By adding “tumour border” features, the machine learning model outperformed the model with “tumour core” regions only, which demonstrated that peri-tumoural tissues contain meaningful features to identify treatment responders. Rectal cancer arises in close association with white adipose tissue (mesorectal fat). Nutrient supply and catabolite drainage to and from the normal rectal wall and rectal tumours must travel through the mesorectal fat by way of vessels and lymphatics^19^, indicating that the mesorectal fat and structures within contain potential predictive information. Jayaprakasam et al. ^33^ extracted radiomics features from mesorectal fat in patients with LARC to predict pathological complete responders (accuracy 83.9%) and local (accuracy 78.3%) or distant recurrence (accuracy 87.0%). Their study further demonstrated the potential predictive value of peri-tumoural regions. MLNet takes not only the peri-tumoural regions but also the global context into consideration. The original MRI was included in the input, which allows the incorporation of global information. In the meantime, local information is highlighted by injecting multi-level feature maps.

In our ablation study, we also observed that feature maps extracted from different stages contributed differently to EMVI classification and CR prediction tasks. The reason might be finer features are more crucial to morphological prognostic factors like EMVI. Conversely, for more challenging and intricate tasks like CR prediction, the integration of multi-level features was more beneficial.

There are some limitations of the study. First of all, despite MLNet outperforming the current state-of-the-art^28^, the sensitivity (0.61) and positive predictive value (PPV) (0.44) were comparatively low, indicating that MLNet’s ability to correctly identify responders was limited, which hinders the implementation of the pipeline in the clinical workflow. The relatively low response rate (around 30%) could be one of the contributors to low sensitivity and PPV. Even though we have applied weighted loss, the data imbalance can still result in limited model performance. To address this issue, Generative Adversarial Networks (GANs) can be used to generate synthetic data for the responder class^34^. Additionally, There is currently no standardised protocol for MRI evaluation of treatment response in locally advanced rectal cancer, which can lead to variability in the labelling of treatment response across different centres^35^. Secondly, all the manual segmentation is based on DWI. T2W was then downsampled in the same domain of DWI, which led to information loss in T2W. Thirdly, the standard of reference for EMVI was based on the assessment by the radiologist using MRI and not pathology considering that patients who underwent CRT and EMVI status post-CRT would no longer be representative of the baseline setting. Fourthly, the dataset in our study was collected over a long time frame from February 2008 and March 2018 from different centres. The significant quality variations have a negative effect on the model performance. We have only used nnUNet pre-processing module to deal with the data heterogeneity. Other state-of-the-art methods can be adopted to deal with data heterogeneity. Modanwal et al. ^36^ have proposed a method based on CycleGAN for MRI normalisation. Their model can successfully learn bidirectional mapping and perform normalisation between MRIs produced by different vendors. Fifthly, although we have the dataset from 9 centres, the total number of samples is only 509. Besides the sample size limitation, the inclusion of a solely Dutch patient cohort may impact the generalisability of the findings. More diverse patient cohorts may be beneficial for this study. Sixthly, some studies^23^ have testified that clinical features like carcinoembryonic antigen (CEA) level could improve the model performance. Collecting CEA and other clinical features could be useful for the MLNet model. Also, integrating other modalities like endoscopic imaging can further enhance the model’s performance. Last but not least important, the retrospective nature of the study is also one of our limitations. A prospective cohort from multiple centres may further demonstrate the performance of the model.

## Methods

### Dataset

Patient data were retrospectively collected if they satisfied the following criterion: (1) biopsy-proven rectal adenocarcinoma; (2) non-metastasised; (3) available pre-treatment MRI (T2W and high *b* value DWI); (4) routine long-course neoadjuvant treatment including radiotherapy total dose 50.0−50.4 Grey with concurrent capecitabine-based chemotherapy; (5) final treatment including surgery or watch and wait with longer than 2 years clinical follow-up. The study was conducted in accordance with the Declaration of Helsinki and has been approved by the Institutional Review Board (IRB) of the Netherlands Cancer Institute. Each participating centre reviewed the study protocol and provided approval. Informed consent was waived by the IRB and by each participating centre during local ethical review and approval due to the retrospective nature of the study. 670 patients were initially collected and 161 patients were excluded see Fig. S1. 509 patients data were obtained using 25 scanners, 94 protocols for DWI and 112 T2W protocols see Table S1. For DWI, b-values range from 600 to 1200. Semi-automatical algorithm using level-tracing was first used to segment all high *b* value DWIs, A board-certified radiologist with >10 years of experience in rectal MRI then manually adjusted the segmentation slice by slice, taking the anatomical information from T2W into consideration, taking care to exclude the rectal lumen and any non-tumour perirectal tissues. The same expert radiologist reported mrEMVI status for each patient. We split patients into training, validation and external testing centre-wise. To have a fair comparison of the CR classification performance with the current state-of-the-art, we kept the same external cohort (3 centres) as Schurink et al. ^28^. Out of the rest 6 centres, 2 centres were randomly chosen as the internal validation.

### Segmentation

nnUNet, proposed by Isensee et al. ^27^, is a deep learning-based segmentation approach that automatically configures itself for any new task, including preprocessing, network design, training, and post-processing. nnUNet has shown great performance over 23 public datasets used in international biomedical segmentation competitions^27^. It has a state-of-the-art prepossessing technique, which automatically generates a dataset fingerprint that contains all relevant parameters and properties. Also, networks are trained with deep supervision strategy^37^. Deep supervision is to provide the supervision of hidden layers and propagate it to lower layers, instead of only supervising at the output layer^38^. In nnUNet, deep supervision downsamples the ground truth masks to different scales with tri-linear interpolation such that it corresponds to the output at each upsampling stage. The final segmenting loss is then the weighted combination of the loss at each of these upsampling stages. Deep supervision allows gradients to be fed deeper into the network and facilitates the training of all layers. All the feature maps at different stages are inferred after segmentation training for further application in classification tasks.

### Classification

The second part of our automated pipeline is a lightweight CNN, which was modified on top of a 3D ResNet10^39^. Other than 3D ResNet10, different backbones were compared in the external cohort and 3D ResNet10 outperformed all other 3D ResNet backbones see Table S11 and Fig. S4. The original MRI was fed into the model as input. Experiments using segmentation features as input without original MRIs underperformed MLNet, see Table S12. Additionally, instead of placing the residual blocks with skip connections, feature maps of different stages inferred from segmentation networks were injected into our classification network as prior knowledge. The feature injection was done by concatenation (Fig. 1b). For the ablation analysis, only the original MRI was used as input for the 3D ResNet10. Single-stage representations were injected into the StageN (*N* = 1, 2, 3, 4) model, with the original MRI serving as the input as well. Multivariate analysis was conducted with logistic regression using the development cohort (412 patients, 6 centres) and external validated with the same data as other models in the ablation analysis.

### Experiment

For the segmentation part, we trained a 4-fold nnUNet and then inferred the predicted masks and corresponding feature maps of 4 stages (from coarse to fine, see Fig. 4 for feature visualization) for both 4-fold validation and external validation. After segmentation training, we split the development data (6 centres, with 412 patients) into a training set (4 centres, 317 patients) and an internal validation set (2 centres, 95 patients). MLNet, as well as other models in the ablation analysis, were constructed using a training cohort, internally validated and further validated on the external validation cohort. The pipeline was constructed using PyTorch^40^. Both nnUNet and MLNet were trained on an NVIDIA RTX 2080 Ti GPU. During the training of nnUNet, all the hyperparameters were automatically configured. During the training of MLNet, the batch size was set to 4 and the initial learning rate was 1e − 4. Weighted binary cross entropy was used as the loss function. Adam^41^ was used as the optimiser. Additionally, shape-aware minimisation (SAM)^42^ simultaneously minimising loss value and loss sharpness was adopted. To avoid overfitting, training patience was set to 10. The best model was saved with the best loss on the internal validation set.

### Statistical analysis

Statistical analysis was performed by using python 3.8.15. For further information, check MLNet github repository. The Dice is used to evaluate tumour segmentation performance. The AUC, sensitivity, specificity, PPV, Negative Predictive Value (NPV) and F1 score are used to evaluate the EMVI classification and CR prediction results. All the metrics are showed in Eqs. 1–7. The operating points for distinguishing between EMVI+ and EMVI-, CR and Non-CR were generated using the maximum Youden index on internal validation cohort and the same threshold was applied on the external set. 95% confidence intervals were generated with bootstrap method with 10,000 replications^43^. The characteristics difference of different cohorts were compared by Kruskal-Wallis Test. Mann–Whitney *U* test was used to compare the difference of indicators among different methods. All statistical analyses were two-sided and *p* value less than 0.05 was regarded as statistically significant. All the metrics in our study are listed as follows:

1 $${Dice}=\frac{2{TP}}{2{TP}+{FP}+{FN}}$$

2 $${AUC}=\frac{{\sum }_{{in}{s}_{i}\in {positiveclass}}{Ran}{k}_{{in}{s}_{i}}-\frac{M* \left(M+1\right)}{2}}{M* N}$$

3 $${Sensitivity}=\frac{{TP}}{{TP}+{FN}}$$

4 $${Specificity}=\frac{{TN}}{{TN}+{FP}}$$

5 $${PPV}=\frac{{TP}}{{TP}+{FP}}$$

6 $${NPV}=\frac{{TN}}{{TN}+{FN}}$$

7 $$F1=\frac{{TP}}{{TP}+\frac{1}{2}\left({FP}+{FN}\right)}$$

Where TP is true positive, FN is false negative and FP denotes false positive. For AUC calculation, M, N are the number of positive samples and negative samples. ${Ran}{k}_{{in}{s}_{i}}$ is the serial number of sample i. ${\sum }_{{in}{s}_{i}\in {positiveclass}}{Ran}{k}_{{in}{s}_{i}}$ is adding up the serial numbers of the positive cases.

### Reporting summary

Further information on research design is available in the Nature Research Reporting Summary linked to this article.

## Supplementary information

Supplementary Information

Reporting Summary

## Source data

Source Data

## Data Availability

The original data is private and is not publicly available to guarantee protection of patients’ privacy. All data supporting the findings can be provided upon reasonable request to the corresponding author for non-commercial and academic purposes. Excel files containing raw data included in the main figures and tables can be found in the Source Data File in the article. We provided all source codes of this study to facilitate reproducibility.

## Code availability

The codes of this paper are available at https://github.com/Liiiii2101/MLNet. It should only be used for academic purposes.
