# Author Correction: An automated deep learning pipeline for EMVI classification and response prediction of rectal cancer using baseline MRI: a multi-centre study

**DOI:** 10.1038/s41698-025-00827-7

**Published:** 2025-02-12

**Authors:** Lishan Cai, Doenja M. J. Lambregts, Geerard L. Beets, Monique Maas, Eduardo H. P. Pooch, Corentin Guérendel, Regina G. H. Beets-Tan, Sean Benson

**Affiliations:** 1 Department of Radiology, The Netherlands Cancer Institute, Plesmanlaan 121, 1066 CX Amsterdam, The Netherlands; 2 GROW School for Oncology and Developmental Biology, Maastricht University Medical Centre, P. Debyelaan 25, 66202 AZ Maastricht, The Netherlands; 3 Department of Surgery, The Netherlands Cancer Institute, Plesmanlaan 121, 1066 CX Amsterdam, The Netherlands

**Keywords:** Cancer imaging, Risk factors

Correction to: *npj Precision Oncology* 10.1038/s41698-024-00516-x, published online 22 January 2024

“In this article, the name of the author Monique Maas was misspelled as Monique Mass. The original article has been corrected.”

