# Supplementary material for: An automated deep learning pipeline for EMVI classification and response prediction of rectal cancer using baseline MRI: a multi-centre study
Source: NPJ Precis Oncol. 2024 Jan 22;8:17. doi: 10.1038/s41698-024-00516-x (PMC10803303; doi:10.1038/s41698-024-00516-x)
Supplement: Supplementary file 1 — Supplementary Information [file 41698_2024_516_MOESM1_ESM.pdf]

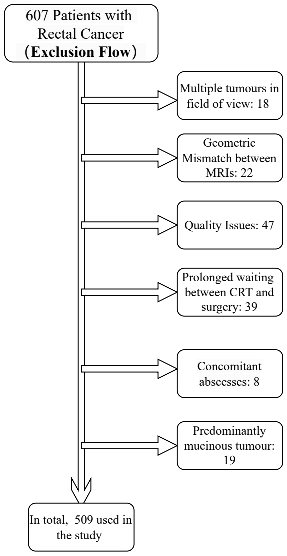

Supplementary Figure 1. Rectal Cancer Patients Exclusion Flowchart. More detailed in- and exclusion criterion can be found in[1].

Supplementary Table 1. MRI Hardwares Overview

Scanners (n)	Scanner model	Vendors			Field Strength		T2 Protocols	DWI Protocols
		Philips	Siemens	GE	1.5T	3.0T		
25	13	10 (6 centers)	12 (12 centers)	3 (2 centers)	19	6	112	94

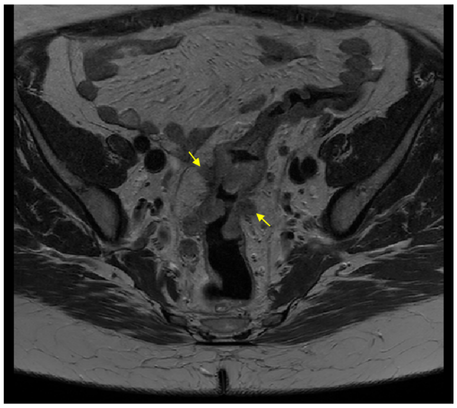

Supplementary Figure 2. The illustration of EMVI for case **III**. yellow arrows, EMVI positive.

Supplementary Table 2. EMVI classification ablation study using DWI+T2W in the external cohort

Network	AUC (95% CI)	Sensitivity(95% CI)	Specificity(95% CI)	PPV (95% CI)	NPV (95% CI)	F1 (95% CI)
Resnet10	0.45 (0.34 - 0.57)	0.47 (0.36 - 0.58)	0.51 (0.40 - 0.62)	0.47 (0.33 - 0.62)	0.50 (0.36 - 0.65)	0.47(0.35 - 0.59)
MLNet	**0.73** (0.62 - 0.83)	**0.63** (0.53 - 0.73)	**0.67** (0.57 - 0.77)	**0.64** (0.50 - 0.77)	**0.66** (0.52 - 0.78)	**0.64**(0.52 - 0.74)
Stage 1	0.71 (0.60 - 0.81)	**0.64** (0.54 - 0.74)	0.68 (0.57 - 0.78)	0.66 (0.50 - 0.79)	0.67 (0.53 - 0.80)	0.65(0.53 - 0.76)
Stage 2	0.57 (0.46 - 0.69)	0.54 (0.42 - 0.65)	0.57 (0.47 - 0.68)	0.53 (0.39 - 0.69)	0.58 (0.43 - 0.71)	0.54(0.41 - 0.66)
Stage 3	0.55 (0.43 - 0.66)	0.50 (0.40 - 0.61)	0.54 (0.44 - 0.65)	0.51 (0.36 - 0.66)	0.54 (0.40 - 0.67)	0.51(0.38 - 0.62)
Stage 4	0.49 (0.38 - 0.61)	0.48 (0.37 - 0.59)	0.52 (0.40 - 0.63)	0.48 (0.33 - 0.63)	0.51 (0.37 - 0.66)	0.48(0.36 - 0.60)

Note: Values in the parentheses were 95% confidence intervals (95% CI). tage1(2,3,4), classification network only infused segmentation features from stage1 (2,3,4). The best metrics were highlighted in bold.

Supplementary Table 3. CR prediction ablation study using DWI+T2W in the external cohort

Network	AUC (95% CI)	Sensitivity(95% CI)	Specificity(95% CI)	PPV (95% CI)	NPV (95% CI)	F1 (95% CI)
Resnet10	0.50 (0.38 - 0.61)	0.47 (0.37 - 0.57)	0.51 (0.40 - 0.61)	**0.48** (0.33 - 0.62)	0.51 (0.37 - 0.65)	0.47(0.35 - 0.59)
MLNet	**0.66** (0.55 - 0.77)	**0.61** (0.49 - 0.72)	**0.65** (0.54 - 0.75)	0.44 (0.28 - 0.60)	**0.79** (0.67 - 0.88)	**0.51**(0.36 - 0.64)
Stage 1	0.57 (0.45 - 0.68)	0.56 (0.44 - 0.67)	0.60 (0.49 - 0.70)	0.38 (0.24 - 0.53)	0.75 (0.63 - 0.85)	0.45(0.32 - 0.58)
Stage 2	0.54 (0.42 - 0.66)	0.47 (0.35 - 0.59)	0.52 (0.41 - 0.62)	0.30 (0.18 - 0.44)	0.69 (0.55 - 0.81)	0.37(0.24 - 0.49)
Stage 3	0.53 (0.41 - 0.66)	0.53 (0.41 - 0.64)	0.58 (0.45 - 0.68)	0.36 (0.22 - 0.51)	0.73 (0.61 - 0.84)	0.43(0.29 - 0.56)
Stage 4	0.59 (0.46 - 0.71)	0.55 (0.42 - 0.67)	0.59 (0.47 - 0.70)	0.37 (0.22 - 0.53)	0.75 (0.62 - 0.85)	0.44(0.30 - 0.58)

Values in the parentheses were 95% confidence intervals (95% CI). tage1(2,3,4), classification network only infused segmentation features from stage1 (2,3,4). The best metrics were highlighted in bold.

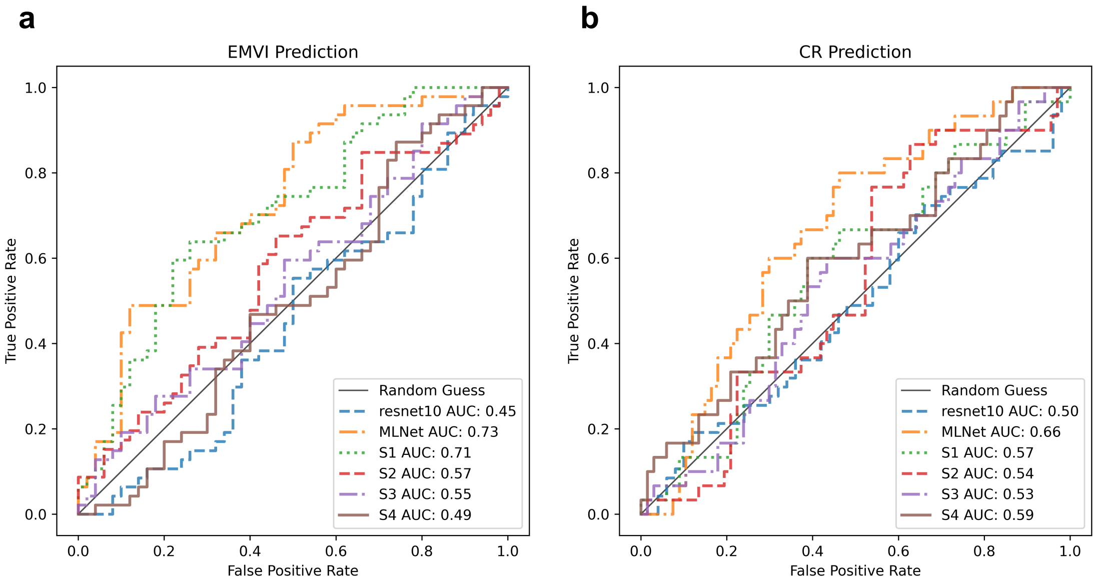

Supplementary Figure 3. The ROC plots of prediction of EMVI (a) and CR (b) using DWI and T2W in the external validation cohort. S1(2,3,4), classification network only infused segmentation features from stage1 (2,3,4)

Supplementary Table 4. EMVI classification ablation study using DWI in the internal cohort

Network	AUC (95% CI)	Sensitivity(95% CI)	Specificity(95% CI)	PPV (95% CI)	NPV (95% CI)	F1 (95% CI)	Threshold
Resnet10	0.54 (0.42 - 0.65)	0.51 (0.40 - 0.62)	0.55 (0.44 - 0.66)	0.46 (0.31 - 0.61)	0.61 (0.47 - 0.74)	0.48 (0.36 - 0.60)	0.41
MLNet	0.76 (0.66 - 0.85)	0.67 (0.56 - 0.76)	0.70 (0.60 - 0.80)	0.62 (0.47 - 0.76)	0.75 (0.62 - 0.84)	0.64 (0.51 - 0.75)	0.42
Stage 1	0.78 (0.68 - 0.87)	0.69 (0.59 - 0.79)	0.73 (0.63 - 0.82)	0.66 (0.50 - 0.79)	0.77 (0.65 - 0.86)	0.67 (0.55 - 0.78)	0.45
Stage 2	0.65 (0.54 - 0.76)	0.60 (0.49 - 0.74)	0.63 (0.53 - 0.74)	0.54 (0.39 - 0.69)	0.68 (0.55 - 0.80)	0.57 (0.44 - 0.69)	0.39
Stage 3	0.55 (0.43 - 0.67)	0.53 (0.41 - 0.64)	0.57 (0.45 - 0.68)	0.47 (0.32 - 0.63)	0.62 (0.48 - 0.75)	0.49 (0.36 - 0.63)	0.45
Stage 4	0.66 (0.54 - 0.76)	0.61 (0.49 - 0.71)	0.64 (0.53 - 0.75)	0.56 (0.40 - 0.70)	0.69 (0.55 - 0.80)	0.58 (0.44 - 0.70)	0.52

Note: values in the parentheses were 95% confidence intervals (95% CI). Stage1 (2,3,4): classification network only infused segmentation features from stage1 (2,3,4). The best metrics were highlighted in bold. The threshold was generated using the maximum Youden index.

Supplementary Table 5. CR prediction ablation study using DWI in the internal cohort

Network	AUC (95% CI)	Sensitivity(95% CI)	Specificity(95% CI)	PPV (95% CI)	NPV (95% CI)	F1 (95% CI)	Threshold
Resnet10	0.54 (0.42 - 0.66)	0.49 (0.37 - 0.61)	0.53 (0.43 - 0.64)	0.38 (0.24 - 0.53)	0.64 (0.50 - 0.77)	0.43 (0.30 - 0.56)	0.47
MLNet	0.62 (0.50 - 0.74)	0.53 (0.40 - 0.65)	0.56 (0.44 - 0.68)	0.41 (0.26 - 0.57)	0.67 (0.52 - 0.80)	0.46 (0.32 - 0.60)	0.37
Stage 1	0.66 (0.54 - 0.77)	0.58 (0.46 - 0.72)	0.61 (0.50 - 0.72)	0.47 (0.31 - 0.62)	0.71 (0.58 - 0.82)	0.51 (0.38 - 0.64)	0.43
Stage 2	0.66 (0.54 - 0.77)	0.55 (0.42 - 0.67)	0.58 (0.47 - 0.71)	0.43 (0.28 - 0.60)	0.69 (0.55 - 0.82)	0.48 (0.35 - 0.62)	0.43
Stage 3	0.59 (0.47 - 0.70)	0.50 (0.37 - 0.62)	0.54 (0.43 - 0.66)	0.39 (0.25 - 0.56)	0.65 (0.51 - 0.77)	0.43 (0.30 - 0.58)	0.45
Stage 4	0.61 (0.49 - 0.72)	0.53 (0.47 - 0.67)	0.57 (0.47 - 0.67)	0.42 (0.28 - 0.57)	0.67 (0.54 - 0.79)	0.47 (0.34 - 0.59)	0.45

Note: values in the parentheses were 95% confidence intervals (95% CI). Stage1 (2,3,4): classification network only infused segmentation features from stage1 (2,3,4). The best metrics were highlighted in bold. The threshold was generated using the maximum Youden index.

Supplementary Table 6. EMVI classification ablation study using DWI+T2W in the internal cohort

Network	AUC (95% CI)	Sensitivity(95% CI)	Specificity(95% CI)	PPV (95% CI)	NPV (95% CI)	F1 (95% CI)	Threshold
Resnet10	0.52 (0.40 - 0.64)	0.51 (0.40 - 0.62)	0.51 (0.40 - 0.61)	0.41 (0.27 - 0.56)	0.57 (0.42 - 0.71)	0.43 (0.32 - 0.56)	0.45
MLNet	0.78 (0.68 - 0.87)	0.72 (0.61 - 0.81)	0.75 (0.65 - 0.84)	0.68 (0.52 - 0.81)	0.79 (0.67 - 0.88)	0.70 (0.57 - 0.80)	0.44
Stage 1	0.79 (0.69 - 0.87)	0.71 (0.60 - 0.80)	0.75 (0.65 - 0.84)	0.67 (0.52 - 0.80)	0.78 (0.66 - 0.87)	0.69 (0.56 - 0.80)	0.42
Stage 2	0.56 (0.44 - 0.68)	0.55 (0.44 - 0.65)	0.59 (0.48 - 0.69)	0.49 (0.35 - 0.64)	0.64 (0.50 - 0.76)	0.52 (0.39 - 0.64)	0.46
Stage 3	0.52 (0.39 - 0.64)	0.51 (0.39 - 0.62)	0.55 (0.43 - 0.66)	0.45 (0.30 - 0.60)	0.60 (0.46 - 0.74)	0.48 (0.34 - 0.60)	0.46
Stage 4	0.54 (0.42 - 0.66)	0.55 (0.44 - 0.66)	0.58 (0.47 - 0.69)	0.49 (0.34 - 0.64)	0.64 (0.50 - 0.77)	0.52 (0.39 - 0.64)	0.49

Note: values in the parentheses were 95segmentation features from stage1 (2,3,4). The best metrics were highlighted in bold. The threshold was generated using the maximum Youden index.

Supplementary Table 7. CR prediction ablation study using DWI+T2W in the internal cohort

Network	AUC (95% CI)	Sensitivity(95% CI)	Specificity(95% CI)	PPV (95% CI)	NPV (95% CI)	F1 (95% CI)	Threshold
Resnet10	0.62 (0.49 - 0.73)	0.59 (0.47 - 0.70)	0.63 (0.51 - 0.74)	0.49 (0.32 - 0.64)	0.73 (0.59 - 0.84)	0.53 (0.39 - 0.66)	0.43
MLNet	0.65 (0.52 - 0.77)	0.58 (0.46 - 0.71)	0.62 (0.50 - 0.75)	0.47 (0.31 - 0.64)	0.72 (0.58 - 0.84)	0.52 (0.38 - 0.67)	0.42
Stage 1	0.60 (0.47 - 0.72)	0.54 (0.42 - 0.65)	0.58 (0.46 - 0.69)	0.42 (0.28 - 0.58)	0.68 (0.54 - 0.80)	0.47 (0.34 - 0.60)	0.45
Stage 2	0.63 (0.50 - 0.75)	0.54 (0.42 - 0.67)	0.58 (0.47 - 0.70)	0.43 (0.29 - 0.59)	0.69 (0.55 - 0.81)	0.48 (0.35 - 0.62)	0.42
Stage 3	0.60 (0.47 - 0.72)	0.52 (0.40 - 0.62)	0.56 (0.45 - 0.66)	0.40 (0.27 - 0.55)	0.66 (0.52 - 0.79)	0.45 (0.33 - 0.58)	0.43
Stage 4	0.67 (0.55 - 0.79)	0.63 (0.52 - 0.74)	0.67 (0.56 - 0.78)	0.53 (0.37 - 0.69)	0.76 (0.63 - 0.86)	0.58 (0.44 - 0.70)	0.42

Note: values in the parentheses were 95segmentation features from stage1 (2,3,4). The best metrics were highlighted in bold. The threshold was generated using the maximum Youden index.

Supplementary Table 8. EMVI classification and CR prediction using Logistic Regression in the training cohort (n = 412)

Network	AUC (95% CI)	Sensitivity(95% CI)	Specificity(95% CI)	PPV (95% CI)	NPV (95% CI)	F1 (95% CI)
EMVI	0.65 (0.59 - 0.70)	0.58 (0.50 - 0.65)	0.64 (0.58 - 0.70)	0.50 (0.43 - 0.57)	0.71 (0.65 - 0.76)	0.53 (0.47 - 0.59)
CR	0.61 (0.55 - 0.67)	0.53 (0.43 - 0.62)	0.61 (0.55 - 0.66)	0.33 (0.26 - 0.40)	0.78 (0.73 - 0.83)	0.41 (0.33 - 0.48)

Note: values in the parentheses are 95% confidence intervals (95% CI).

Supplementary Table 9. EMVI Classification AUCs in the training cohort (n = 317)

Network	ResNet10	MLNet	Stage 1	Stage 2	Stage 3	Stage 4
DWI	0.85 (0.81 - 0.89)	0.76 (0.71 - 0.82)	0.76 (0.71 - 0.82)	0.73 (0.67 - 0.79)	0.74 (0.68 - 0.79)	0.63 (0.57 - 0.70)
DWI+ T2	0.87 (0.82 - 0.90)	0.83 (0.78 - 0.88)	0.78 (0.73 - 0.83)	0.77 (0.72 - 0.82)	0.73 (0.67 - 0.78)	0.71 (0.65 - 0.76)

Note: values in the parentheses are 95% confidence intervals (95% CI).

Supplementary Table 10. CR Prediction AUCs in the training cohort (n =317)

Network	ResNet10	MLNet	Stage 1	Stage 2	Stage 3	Stage 4
DWI	0.83 (0.77 - 0.87)	0.79 (0.73 - 0.84)	0.57 (0.50 - 0.64)	0.75 (0.68 - 0.81)	0.65 (0.58 - 0.72)	0.72 (0.65 - 0.78)
DWI+ T2	0.82 (0.76 - 0.87)	0.64 (0.57 - 0.71)	0.74 (0.67 - 0.80)	0.75 (0.68 - 0.81)	0.64 (0.56 - 0.71)	0.66 (0.58 - 0.73)

Note: values in the parentheses are 95% confidence intervals (95% CI).

Supplementary Table 11. EMVI classification and CR predictions AUCs with DWI alone using different ResNet backbones in the external cohort

Backbones	ResNet10	ResNet18	ResNet34	ResNet50	ResNet101
EMVI	**0.76** (0.66 - 0.84)	0.74 (0.63 - 0.83)	0.47 (0.35 - 0.59)	0.53 (0.41 - 0.65)	0.48 (0.36 - 0.59)
CR	**0.62** (0.49 - 0.73)	0.48 (0.36 - 0.60)	0.41 (0.29 - 0.54)	0.47 (0.35 - 0.59)	0.44 (0.32 - 0.56)

Values in the parentheses are 95% confidence intervals (95% CI). ResNetx, MLNet was built on top of the backbone ResNetx.

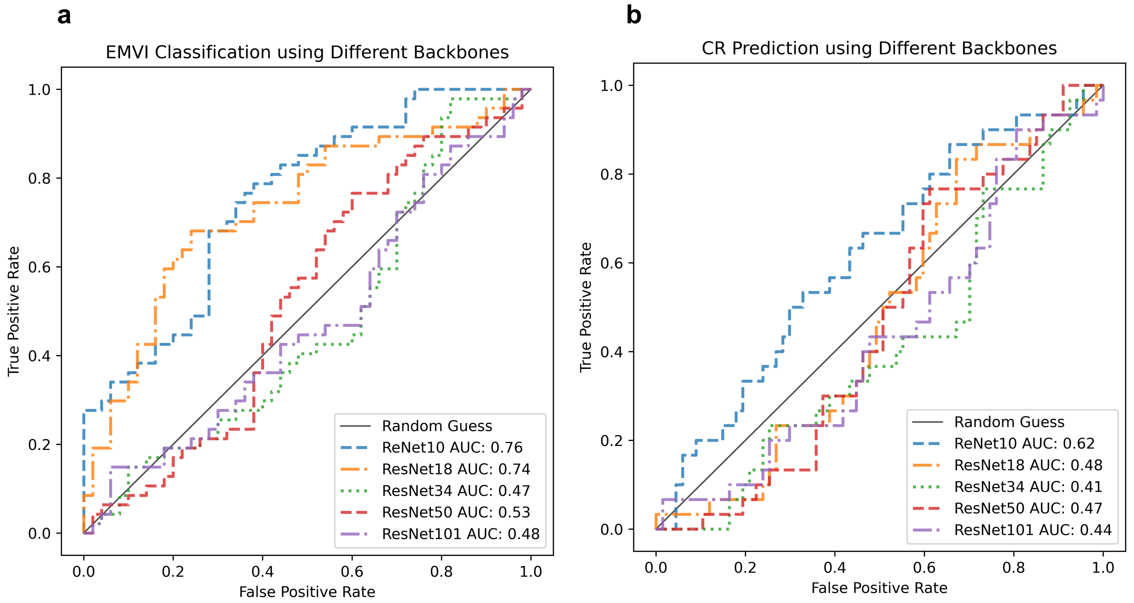

Supplementary Figure 4. The ROC plots of prediction of EMVI classification (a) and CR prediction (b) using DWI on external validation cohort with MLNet using different ResNet backbones including ResNet10, ResNet18, ResNet34, ResNet50 and ResNet101.

Supplementary Table 12. EMVI and CR classification AUCs with different inputs using 3D ResNet10 in the external cohort

Inputs	Predicted Tumour	FM-Stage1	FM-Stage2	FM-Stage3	FM-Stage4
EMVI	0.71 (0.60 - 0.81)	0.71 (0.60 - 0.80)	0.54 (0.42 - 0.65)	0.52 (0.40 - 0.64)	0.52 (0.40 - 0.64)
CR	0.56 (0.44 - 0.68)	0.55 (0.42 - 0.68)	0.55 (0.42 - 0.68)	0.52 (0.39 - 0.66)	0.50 (0.37 - 0.62)

Values in the parentheses are 95% confidence intervals (95% CI). Predited Tumour: predicted tumour as input.
FM-Stage1(2,3,4), feature maps from stage1(2,3,4) as input

Segmentation Loss:
if we denote the weights of different stages (1-4) of nnUNet as:

$$\mathbf{W} = (W^{(1)}, W^{(2)}, W^{(3)}, W^{(4)}) \tag{1}$$

W^{i}_{out} denotes the output feature maps at i stage for sample x_j

$$W^{i}_{out\,j} = x_j; \mathbf{W}, W^{i} \tag{2}$$

the segmentation loss at stage i can be expressed as:

$$L^{i}_{seg}(X; \mathbf{W}, \mathbf{w}^{i}) = \sum_{x_j \in X} (-\log p(y_{seg\,j}) | W^{i}_{out}) + 1 - \frac{2y_j W^{i}_{out} + 1}{y_j + W^{i}_{out}}) \tag{3}$$

The final segmentation loss is :

$$L_{seg} = \alpha_0 L^{final}_{seg} + \alpha_1 L^{1}_{seg} + \alpha_2 L^{2}_{seg} + \alpha_3 + L^{3}_{seg} + \alpha_4 L^{4}_{seg} \tag{4}$$

hereby the loss weights α are halved with each decrease in resolution, $\alpha_4 = \frac{1}{2}\alpha_3 = \frac{1}{4}\alpha_2 = \frac{1}{8}\alpha_2 = \frac{1}{16}\alpha_1 = \frac{1}{32}\alpha_0$

MLNet Loss: For the classification networks (MLNet), at different stages in MLNet, the features learned by the nnUNet model will be injected by concatenation operation, resulting in the following total training loss for MLNet :

$$L_{cls}(X, W^{1}_{out}, W^{2}_{out}, W^{3}_{out}, W^{4}_{out}; \mathbf{W}_{cls}) = \sum_{x_j \in X} -\log p(y_{cls\,j} | x_j, W^{1}_{out\,j}, W^{2}_{out\,j}, W^{3}_{out\,j}, W^{4}_{out\,j}; \mathbf{W}_{cls}) \tag{5}$$

References

1. Schurink, N. W. *et al.* Development and multicenter validation of a multiparametric imaging model to predict treatment response in rectal cancer. *Eur. Radiol.* 1–10 (2023).
